# Puffing of Medical Professors, and the Arts of Quackery

**Published:** 1850-11

**Authors:** 


### PUFFING OF MEDICAL PROFESSORS, AND THE ARTS OF QUACKERY.

We intended to say something on this subject, but there is so much truth, so much good sense, in the following remarks of a correspondent of the *Boston Medical and Surgical Journal*, that we prefer republishing them, rather than anything of our own. We had marked the exceptional matter in the *New York Tribune*, upon which "Honestus" animadverts severely, and intended to base our exceptions to some professorial arts in the self-eulogistic line, upon the description of Professor Cox's lecture given the *Tribuue*, but "Honestus" has done ample justice to the subject, and we give way to him.

### "QUACKERY IN THE REGULAR PROFESSON.

*"(Communicated for the Boston Medical and Surgical Journal.)*

"In an article in a recent issue of the Boston Medical and Surgical Journal, the writer alludes to certain cliques, said to be in New York, who make it a point to sustain their members, above and to the exclusion of all others. Every high-minded physician must coincide with the views there expressed. Nearly allied to this practice is that of some physicians, either directly or indirectly sounding their individual praises. Various are their methods. They have, for every ear, some most wonderful case to relate, which has fallen under their care, and which invariably terminates successfully. Or, they have been called to such and such well-known individuals, who, the auditor is aware, have previously embloyed another physician. Or, they vaunt themselves of the number of their cases, and their consultations, and are ever in hot haste, pressing calls preventing a moment's delay. Or, they have been called to such an aggravated case, describing minutely and graphically the intense agonies of the sufferer, and how entirely and quickly *he* relieved him. Others, still, seek popularity by underbidding in price. He is "the poor man's doctor;" "the other physicians charge exhorbitantly," &c., &c. These are but a few, of the various ways, to which individuals resort, to talk themselves into notoriety. The reader will readily recall one or more such persons, who study these various methods, and practise them, quite as much as physic or surgery. This is nothing more nor less than sheer quackery. The most disreputable charletan does the same. Will a noble, high-minded physician stoop to such practices? He is conscious of worth, of rectitude, of talent, and he goes forth into the world, confident that true merit will be slowly, but surely, appreciated. He preserves his dignity, his self-respect, and his would be the last voice to utter a word in self-commendation.

"This immodest self-praise has even been heard from the professors in our medical schools, in the character of their circulars, and the publishing rather conspicuously the number of students, of graduates, &c., &c. In a late New York Tribune, is an article giving an account of the opening of the New York Medical College, in which the speech of Dr. Cox, Professor of Surgery in this College, was spoken of as "the principal feature of the evening. The writer says, "It was a splendid and elegant production. He pronounced a detailed eulogy upon his coadjutors in the professiorial chairs," &c. &c. I had hoped to have seen the above extract contradicted, supposing the statement incorrect. I could with difficulty believe, that the professors of that school would appoint one of their own number to appear before an audience, to pronounce a "detailed eulogy" upon themselves. Many other things of a like nature might be mentioned. "These things ought not so to be." The medical profession should not be degraded by such dishonorable practices. Let every member of the profession, who has its good at heart, speak out in censure of these abuses as they justly deserve. Would every physician be what he desires the world to believe him to be, he would no longer be obliged to resort to such means of self-glorification. HONESTUS."

These are crying evils and should be promptly checked. There is certainly enough high-toned feeling in a learned and liberal profession, enough sound, discriminating judgment in the community to curb the wrongs of which we complain. There may be justice in the plea sometimes made that if a man cannot afford to pay a trumpeter, he must blow for himself, but we think he might have the grace to wait until he has a trumpet of his own to blow. B.

